# Aumento de Óbitos Domiciliares devido a Parada Cardiorrespiratória em Tempos de Pandemia de COVID-19

**DOI:** 10.36660/abc.20200547

**Published:** 2021-02-19

**Authors:** Nathalia Sernizon Guimarães, Taciana Malheiros Lima Carvalho, Jackson Machado-Pinto, Roger Lage, Renata Mascarenhas Bernardes, Alex Sander Sena Peres, Mariana Amaral Raposo, Ricardo Machado Carvalhais, Renan Avelino Mancini, Gabriella Yuka Shiomatsu, Bruna Carvalho Oliveira, Valéria de Melo Rodrigues, Maria do Carmo Barros de Melo, Unaí Tupinambás

**Affiliations:** 1 Universidade Federal de Ouro Preto Ouro PretoMG Brasil Universidade Federal de Ouro Preto, Ouro Preto, MG - Brasil; 2 Prefeitura Municipal de Belo Horizonte Belo HorizonteMG Brasil Prefeitura Municipal de Belo Horizonte, Belo Horizonte, MG - Brasil; 3 Universidade Estadual de Campinas CampinasSP Brasil Universidade Estadual de Campinas, Campinas, SP - Brasil; 4 Universidade Federal de Minas Gerais Belo HorizonteMG Brasil Universidade Federal de Minas Gerais, Belo Horizonte, MG - Brasil

**Keywords:** COVID-19, Betacoronavírus, Pandemia, Parada Cardíaca, Óbitos, Serviços Médicos de Emergência

## Abstract

**Fundamento:**

As doenças cardiovasculares constituem um grupo importante de causas de morte no Brasil. As doenças isquêmicas do coração são as principais causas de parada cardiorrespiratória, levando a um impacto na mortalidade devido às doenças cardiovasculares no sistema de saúde.

**Objetivo:**

Avaliar o número de óbitos domiciliares por parada cardiorrespiratória notificados pelo Serviço de Atendimento Móvel de Urgência (SAMU) em março de 2018, 2019 e 2020.

**Métodos:**

Trata-se de um estudo observacional realizado a partir da análise de dados de mortalidade por parada cardiorrespiratória de cidadãos atendidos pelo SAMU em Belo Horizonte, Minas Gerais, Brasil. Foram analisadas as características sociais e clínicas e as informações de ocorrência. Foi avaliada a taxa de mortalidade por parada cardiorrespiratória em relação ao número total de atendimentos. Foi considerado um nível de significância de 95%.

**Resultados:**

Houve um aumento nos óbitos domiciliares por parada cardiorrespiratória em março de 2020, em comparação com março de 2018 (p < 0,001) e março de 2019 (p = 0,050). Dos óbitos relatados em 2020, 63,8% dos pacientes tinham 60 anos ou mais; 63,7% das ocorrências foram à tarde e aproximadamente 87% dos casos de parada cardiorrespiratória notificados apresentavam comorbidades clínicas, com hipertensão arterial sistêmicas e parada cardíaca correspondendo a 22,87% e 13,03% dos casos relatados, respectivamente. A maioria da amostra avaliada deste estudo não teve acompanhamento médico (88,7%).

**Conclusão:**

Considerando o aumento do número de óbitos, sugerimos reflexões e reajustes quanto ao monitoramento das doenças crônicas não transmissíveis durante a pandemia, bem como melhorias na vigilância dos óbitos. (Arq Bras Cardiol. 2021; 116(2):266-271)

## Introdução

O Serviço de Atendimento Móvel de Urgência (SAMU) representa o componente móvel de emergência normativamente instituído pelo Sistema Único de Saúde (SUS). Integrante da Rede de Urgência e Emergência desde 2003, o SAMU é atualmente um serviço público de assistência que atua com o objetivo de atendimento pré-hospitalar móvel no SUS. Além disso, este serviço transporta pacientes para hospitais privados, sendo um importante componente da admissibilidade de pacientes da rede privada de saúde.^1 , 2^

O SAMU é composto por centrais de regulação e equipes de ambulâncias, compostas por médicos e enfermeiras. De acordo com as diretrizes preconizadas pelo SUS, qualquer cidadão pode solicitar atendimento móvel pré-hospitalar por meio do acesso gratuito ao telefone, ligando para o número 192. Na central de regulação, um operador de telefone identifica o paciente e o local da ligação e transfere o atendimento ao médico regulador, que pode orientar o paciente por telefone ou ligar para a equipe de assistência para atender à solicitação do usuário. Todas as etapas do atendimento são registradas com o consentimento de ambas as partes, profissionais e usuários.^1^

As equipes de ambulâncias são compostas por unidades de suporte básico, nas quais estão presentes um técnico de enfermagem, motoristas e enfermeiros, e unidades de suporte avançado, uma ambulância com mais recursos tecnológicos e a presença de médico e enfermeiro. A depender das necessidades regionais, as ambulâncias são caracterizadas por motocicletas, barcos ou sistema aeromédico composto por helicóptero ou avião.^3^

As doenças cardiovasculares (DCV) constituem atualmente um importante grupo de causas de óbito no Brasil e no mundo. De acordo com a Sociedade Brasileira de Cardiologia, até o primeiro dia de julho, as DCV causaram mais de 198.000 óbitos entre os brasileiros em 2020.^4^ Estas doenças incluem as doenças isquêmicas do coração, que são as principais causas de parada cardiorrespiratória (PCR).

Segundo o Cardiômetro, indicador do número de óbitos por DCV, criado pela Sociedade Brasileira de Cardiologia, entre 2004 e 2014, as doenças isquêmicas constituíram o grupo de causas cardiovasculares com maior prevalência de eventos de óbito por DCV.^4^ Os dados na literatura a respeito da incidência de PCR no Brasil são escassos, sendo observado o impacto desse evento na mortalidade dos indivíduos.^5 - 7^

Neste contexto, este estudo tem como objetivo descrever o número de óbitos domiciliares por PCR notificados pelo SAMU no município de Belo Horizonte em 2020 e comparar os óbitos domiciliares por PCR em março de 2020 em relação a março de 2018 e março de 2019.

## Métodos

Este estudo faz parte do serviço de notificação do SAMU de Belo Horizonte, Minas Gerais, Brasil, e se refere aos dados coletados em março de 2018, março de 2019 e março de 2020. As notificações foram selecionadas a partir do processamento manual de fichas referentes ao atendimento total das equipes no período previamente determinado. Não foram estabelecidos critérios de exclusão para avaliação de usuários/óbitos. Assim, a amostragem foi realizada por conveniência, contemplando todas as notificações cadastradas no serviço nos períodos descritos.

Trata-se de um estudo observacional retrospectivo, realizado a partir da análise de dados primários de mortalidade por PCR em cidadãos atendidos pelo SAMU em Belo Horizonte.

### Análise Estatística

Foram coletadas a idade, as características de ocorrência (dia do mês e hora do dia) e as características clínicas (causa da PCR, acompanhamento médico e comorbidades associadas) e foi calculada a taxa de mortalidade de acordo com o sistema de notificação do SAMU.

Os dados foram coletados pelos pesquisadores do serviço e, subsequentemente, submetidos à análise descritiva. A análise descritiva das variáveis foi realizada por meio da distribuição de frequências e números absolutos das variáveis categóricas. Foram estimados a prevalência dos desfechos e os intervalos de confiança de 95% para a população.

Para análise dos dados, foi utilizado o programa estatístico público e gratuito OpenEpi®. As variáveis categóricas foram analisadas por meio da distribuição de frequência e comparadas pelo teste qui-quadrado. O nível de significância foi definido em 95% (p < 0,05)

## Resultados

Foram registrados 1.662 óbitos pelo SAMU nos meses de março de 2018 (n = 563), 2019 (n = 494) e 2020 (n = 605). Nesse período (março), o SAMU registrou 919 óbitos domiciliares por PCR nos anos de 2018, 2019 e 2020, distribuídos em 260 óbitos em março de 2018 (28,3%), 283 óbitos em março de 2019 (30,8%) e 376 óbitos em março de 2020 (40,9%). Observou-se que as taxas de óbito para o número total de atendimentos no SAMU nesses períodos foram 0,51, 0,57 e 0,62, respectivamente.

**Figura 1:**
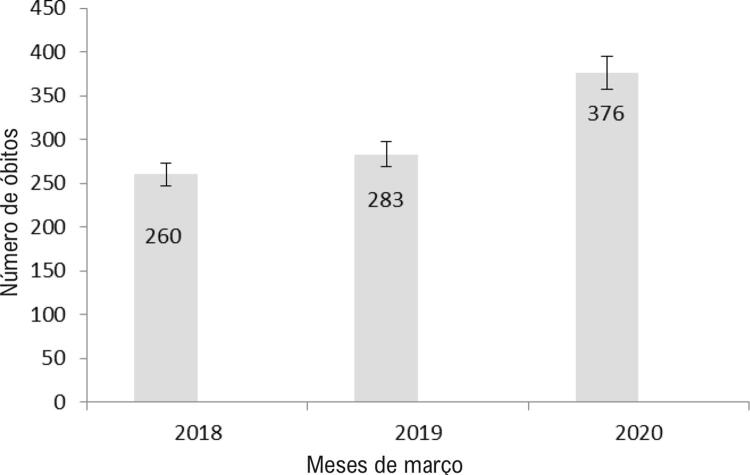
– Prevalência do desfecho de óbito em pacientes atendidos pelo Serviço de Atendimento Móvel de Urgência de Belo Horizonte, Minas Gerais, Brasil, Março 2018-2020.

A Figura 1 mostra um aumento de 33% nos casos de óbitos domiciliares entre março de 2018 e março de 2020. A Tabela 1 compara o número bruto de óbitos por PCR e outras causas, mostrando que 2020 teve mais notificações de óbitos domiciliares por PCR, com diferença estatística em relação a 2018 e 2019.

**Tabela 1:** – Comparação de óbitos domiciliares por parada cardiorrespiratória e outras causas em março de 2018 e 2019 em relação a março de 2020, notificados pelo Serviço de Atendimento Móvel de Urgência de Belo Horizonte, Minas Gerais, Brasil

Óbitos	Anos		Valor p *	Anos		Valor p *
	2018	2020		2019	2020	
PCR	260	376	< 0,01	283	376	0,05
Outras causas	303	229		211	229	

PCR: parada cardiorrespiratória; valor p* - teste qui-quadrado.

A Tabela 2 descreve as características sociais, clínicas e de notificação. Dos óbitos registrados em março de 2020, 63,8% dos pacientes tinham 60 anos ou mais. De acordo com o horário de notificação, 63,7% ocorreram no período diurno sendo 37,7% no período da manhã e 26,0% no período da tarde, sem diferença entre as proporções dos 31 dias consecutivos desse período ( Tabela 2 ).

**Tabela 2:** – Análise descritiva de óbitos por parada cardiorrespiratória em março de 2020, notificados pelo Serviço de Atendimento Móvel de Urgência de Belo Horizonte, Minas Gerais, Brasil

Variáveis	n	%
**Características sociais **		
**Idade (anos)**		
≤ 19	10	2,70
20-59	126	33,50
≥ 60	240	63,80
**Características de ocorrência de SAMU **		
**Atendimento**		
Primeira metade de março	190	50,50
Segunda metade de março	186	49,50
Tempo de ocorrência de SAMU		
06:00 – 11:59	142	37,70
12:00 – 17:59	98	26,00
18:00 – 23:59	89	23,60
00:00 – 05:59	47	12,70
**Características clínicas**		
**Causa de CPA**		
Clínica	326	86,70
Trauma	50	13,30
**Acompanhamento médico **		
Sim	45	12,00
Não	331	88,00

*PCR: parada cardiorrespiratória; SAMU: Serviço de Atendimento Móvel de Urgência. Tamanho da amostra = 376.*

Em relação às características clínicas, observou-se que aproximadamente 87,0% dos pacientes com PCR apresentavam outras comorbidades clínicas e que a maioria da amostra avaliada não teve acompanhamento médico de acordo com os dados coletados (88,7%) ( Tabela 2 ). No entanto, muitos dos acompanhantes e familiares dos pacientes desconheciam o histórico dos pacientes.

A Tabela 3 descreve as comorbidades clínicas notificadas pelo serviço, que representam 87,0% (n = 331) dos pacientes avaliados, de acordo com a evolução da doença observada. Dentre as doenças crônicas observadas, a hipertensão esteve associada à PCR em 22,87% dos casos notificados; a insuficiência cardíaca estava presente em 13,03% dos casos e a diabetes mellitus em 11,0%. Dentre outras comorbidades, é interessante notar que em 38,4% dos casos relatados, apesar de a família ou os amigos relatarem a presença de comorbidade associada, eles não souberam informar quais eram as comorbidades associadas do paciente.

**Tabela 3:** – Comorbidades relacionadas a óbitos por parada cardiorrespiratória em março de 2020, notificados pelo Serviço de Atendimento Móvel de Urgência de Belo Horizonte, Minas Gerais, Brasil

Comorbidades clínicas	n	%
Não relatadas	145	38,56
Hipertensão arterial	86	22,87
Insuficiência cardíaca	49	13,03
Diabetes mellitus	42	11,17
Câncer	40	10,64
Demência	26	6,91
Infecções respiratórias	23	6,12
Acidente vascular cerebral	19	5,05
Arritmia	12	3,19
Infecção do trato urinário	3	0,80

*Tamanho da amostra = 376.*

## Discussão

Como principal resultado deste estudo, observou-se aumento numérico gradativo da taxa de óbitos domiciliares por PCR para o total de atendimentos pelo SAMU e aumento proporcional de 33% dos óbitos domiciliares em março de 2020, mês em que a Organização Mundial da Saúde declarou a pandemia de COVID-19.^8^

Desde a confirmação do primeiro caso no Brasil, em 26 de fevereiro de 2020, a imprensa, assim como as autoridades sanitárias, na ausência de vacinas ou medicamentos antivirais, alertam sobre a necessidade de distanciamento social, uso de máscaras, lavagem das mãos e reforço para cuidados com relação à etiqueta respiratória. Na cidade de Belo Horizonte, a primeira notificação de COVID-19 ocorreu em 16 de março de 2020; entretanto, desde a declaração mundial da pandemia, a prefeitura instituiu o isolamento social precocemente, evitando que os residentes tivessem contato não essencial.

Aproximadamente 80% dos casos de COVID-19 são leves ou oligossintomáticos.^9^ Um estudo recente mostra que 20% de pacientes necessitaram de cuidados hospitalares, com 5% a 15% destes sendo tratados em unidades de terapia intensiva com necessidade de suporte ventilatório. A mortalidade para estes pacientes pode chegar a 80%.^9^O SARS-CoV-2 pode ser transmitido de pessoa para pessoa (contato com as mãos, tosse, ou gotículas de saliva) ou através de superfícies e objetos contaminados pelo vírus.^10^ Em 16 de maio de 2020, mais de 4.605.673 pessoas foram diagnosticadas com COVID-19 e houve 310.180 mortes ao redor do mundo. No Brasil, até 16 de maio de 2020, foram registrados 222.877 casos, com 15.046 óbitos.^11^ Dentre as medidas de prevenção da infecção pela COVID-19, recomenda-se aos indivíduos a higienização das mãos, superfícies e objetos com água e sabão ou com desinfetante para as mãos. Outras indicações incluem evitar o contato com os olhos, nariz e boca; o uso permanente de máscara facial; a manutenção de hábitos saudáveis de alimentação e sono; e o isolamento e distanciamento social.^12^

O aumento do número de mortes, no contexto da pandemia, pode agravar o medo dos usuários de sair do isolamento social para buscar assistência médica e serviços essenciais. Isso poderia atrasar a demanda por serviços de saúde que afetam a doença subjacente. É interessante notar que quase 89% da nossa amostra não teve acompanhamento médico; em frequência semelhante (87%), a natureza da PCR foi a causa clínica.

Em um estudo recente, Gonzales-Olmo et al.,^13^ com dados da população de Madrid, demonstrou elevados níveis de auto-percepção individual de maior vulnerabilidade em relação à infecção pela COVID-19 ao procurar atendimento odontológico, considerado um serviço essencial de saúde. Assim, os pesquisadores observaram que a amostra de indivíduos com mais de 60 anos com doenças sistêmicas evitou o atendimento odontológico na maioria das vezes.^13^

De acordo com os nossos resultados, Holmes et al. (2020) indicaram que houve uma quebra estrutural nas séries temporais de internações semanais diferidas anualmente de acordo com os serviços de emergência no Reino Unido entre setembro de 2019 e abril de 2020.^14^ Esses pesquisadores observaram o período de tempo correspondente a setembro de 2019 e abril de 2020.

Foram desenvolvidas e estão em fase de validação escalas para avaliar o medo dos indivíduos (como a “Escala de Medo de COVID-19”) com a finalidade de avaliar essa emoção. Espera-se que novos estudos avaliem o contexto da pandemia a esse respeito, monitorando o medo em detrimento de não procurar serviços essenciais de saúde, melhorando a qualidade de vida e retardando a mortalidade por causas que não envolvem COVID-19.^15 - 17^

Julia et al.,^18^ relatam a reorganização do serviço de saúde na França durante a pandemia. Importantes investimentos têm sido feitos em termos de teleconsultas para acompanhamento de pacientes com COVID-19, mas a população mais vulnerável com dificuldades de acesso à internet e tecnologias digitais ou com barreiras linguísticas permaneceu sem assistência adequada. Além disso, os leitos hospitalares disponíveis foram drasticamente reduzidos e os profissionais de atenção primária à saúde consequentemente tiveram que lidar com emergências por doenças crônicas.^18^ As visitas domiciliares têm sido divididas por pacientes com e sem COVID-19, levando à sobrecarga do serviço. Já foi adotado um fluxo para a atenção básica aos pacientes com doenças crônicas, a fim de evitar atrasos no controle ambulatorial.^17^

Em Belo Horizonte, houve redução da demanda da população por atendimento nas Unidades Básicas de Saúde e Unidades de Pronto Atendimento. Durante os primeiros quatro meses de 2019, foram atendidas 1.478.905 pessoas; no mesmo período de 2020, esse número foi de 1.215.543, o que representa uma redução de 18%.^19^ Dessa maneira, é importante monitorar o manejo das doenças crônicas e promover ações educativas para que a população compreenda a importância do acompanhamento da saúde e do uso regular dos medicamentos necessários. Dessa forma, a Secretaria Municipal de Saúde de Belo Horizonte atualizou o fluxo de atenção na atenção básica.^19 , 20^

Souza et al.,^21^ relatam sobre a atenção primária à saúde prestada pelo SUS no Brasil e sobre os investimentos que vêm sendo destinados à aquisição de equipamentos e à ampliação de leitos hospitalares para pacientes com COVID-19. Reforçam a necessidade de fortalecer a atenção básica como instrumento de prevenção do colapso do sistema de saúde, evitando óbitos por COVID-19 e doenças crônicas.^21^ É necessário refletir sobre como encontrar o equilíbrio para que as ações de saúde não sejam paralisadas durante a pandemia.

A Secretaria Municipal de Saúde de Belo Horizonte tem realizado ações como treinamentos por meio de conferências virtuais, aulas virtuais, discussão de casos clínicos e notas técnicas para atendimento e fluxo de pacientes com COVID-19, estabelecendo parcerias para atendimento online e atendimento a pacientes de grupos de risco. Paralelamente, vem adotando medidas de adequação dos serviços de urgência e atenção primária à saúde, buscando não sobrecarregar os serviços e promover melhor atendimento aos pacientes com e sem COVID-19.^20^

O aumento do número de óbitos domiciliares tem despertado a atenção de gestores de saúde, e a ideia é reforçar a importância do controle ambulatorial das doenças crônicas e esclarecer as medidas de segurança adotadas para a população quanto às complicações clínicas não relacionadas à COVID-19 durante a pandemia.

Este é um momento favorável para reinventar a relação entre os usuários e os profissionais da atenção básica, buscando maior aproximação para o estabelecimento de vínculos, fortalecendo a importância do controle das doenças crônicas e evitando mortes desnecessárias. Também é necessário implementar medidas para melhorar a vigilância de óbitos. Os serviços de atenção primária à saúde devem organizar a assistência e os fluxos de, pacientes a fim de garantir o atendimento adequado aos pacientes com e sem COVID-19.

Como limitação do estudo, apontamos o alto percentual de indivíduos para os quais não foi possível coletar informações sobre a existência de comorbidades (38,5% da amostra) devido à falta de informações dos familiares ou amigos entrevistados, que podem ter subestimado os valores apresentados.

## Conclusão

Os resultados indicam um aumento no número de óbitos domiciliares por PCR notificados pelo SAMU em março de 2020 em relação a março de 2018 e março de 2019 em Belo Horizonte, Minas Gerais. Será necessário realizar estudos futuros com delineamento longitudinal para monitorar o aumento da mortalidade de usuários do sistema de saúde e analisar suas relações causais para evitar óbitos por outras doenças que não envolvam a COVID-19.
