# Supplementary material for: Aumento de Óbitos Domiciliares devido a Parada Cardiorrespiratória em Tempos de Pandemia de COVID-19
Source: Arq Bras Cardiol. 2021 Feb 19;116(2):266–71. [Article in Portuguese] doi: 10.36660/abc.20200547 (PMC7909962; doi:10.36660/abc.20200547)
Supplement: Translation

# Translation: Increased Home Death Due to Cardiopulmonary Arrest in Times of COVID-19 Pandemic

**Authors:** Nathalia Sernizon Guimarães, Taciana Malheiros Lima Carvalho, Jackson Machado-Pinto, Roger Lage, Renata Mascarenhas Bernardes, Alex Sander Sena Peres, Mariana Amaral Raposo, Ricardo Machado Carvalhais, Renan Avelino Mancini, Gabriella Yuka Shiomatsu, Bruna Carvalho Oliveira, Valéria de Melo Rodrigues, Maria do Carmo Barros de Melo, Unaí Tupinambás

**Affiliations:** 1 Brasil Universidade Federal de Ouro Preto, Ouro Preto, MG - Brasil; 2 Brasil Prefeitura Municipal de Belo Horizonte, Belo Horizonte, MG - Brasil; 3 Brasil Universidade Estadual de Campinas, Campinas, SP - Brasil; 4 Brasil Universidade Federal de Minas Gerais, Belo Horizonte, MG - Brasil

**Keywords:** COVID-19, Betacoronavirus, Pandemics, Heart Arrest, Deaths, Emergency Medical Services

## Abstract

**Background:**

Cardiovascular diseases constitute an important group of causes of death in the country. Ischemic heart diseases that are the main causes of cardiopulmonary arrest, leading to an impact on the mortality of the cardiovascular diseases in the health system.

**Objective:**

Assess the number of home deaths by cardiopulmonary arrest notified by the Mobile Emergency Medical Service (SAMU) in March 2018, 2019 and 2020.

**Methods:**

Observational study carried out from the analysis of cardiopulmonary arrest mortality data of citizens assisted by SAMU in Belo Horizonte, Minas Gerais, Brazil. Social and clinical characteristics and occurrence information of the patients were analyzed. The mortality rate due to cardiopulmonary arrest in relation to the total number of attendances was assessed. A significance level of 95% was considered.

**Results:**

There was increase of home deaths due to cardiopulmonary arrest in March 2020 compared to March 2018 (p<0.001) and March 2019 (p=0.050). Of the deaths reported in 2020, 63.8% of the patients were aged 60 years or older, 63.7% of the occurrences were performed in the afternoon and approximately 87% of the cardiopulmonary arrest notified had associated clinical comorbidities, with systemic arterial hypertension and heart failure represented by 22.87% and 13.03% of the reported cases, respectively. The majority of the evaluated sample of this study did not have any medical care follow-up (88.7%).

**Conclusion:**

Considering the increase in the number of the deaths, we suggest reflections and readjustments regarding the monitoring of chronic non-transmissible diseases during a pandemic, as well as improvements in death surveillance. (Arq Bras Cardiol. 2021; 116(2):266-271)

## Introduction

Mobile Emergency Care Service (SAMU) represents the mobile emergency component that is normatively instituted by the Brazilian Public Health System (SUS). A component of the Urgency and Emergency Network since 2003, SAMU is currently a public assistance service working with the objective of mobile pre-hospital assistance in SUS. In addition, this service brings patients to private hospitals, and it is an important component of admissibility of patients from the private healthcare network.^1 , 2^

SAMU comprises central regulation and ambulance teams, composed of doctors and nurses. According to the guidelines recommended by SUS, any citizen can request pre-hospital mobile assistance through free telephone access by calling the number 192. At central regulation, a telephone operator identifies the patient and the location of the call and transfers the service to the medical regulator, who can guide the patient by phone or call the assistance team to answers the user’s request. All stages of care are recorded with the consent of both parties, professionals and users.^1^

The ambulance teams consist of basic support units, in which a nursing technician, drivers and nurses are present, and advanced support units, an ambulance with more technological resources and the presence of a doctor and a nurse. Depending on regional needs, the ambulances are motorcycles, boats, or an aeromedical system consisting of a helicopter or airplane.^3^

Cardiovascular diseases (CVD) are currently an important group of causes of death in Brazil and worldwide. According to the Brazilian Society of Cardiology, as of the first day of July, CVD caused more than 198,000 deaths among Brazilians in 2020.^4^These diseases include ischemic heart diseases, which are the main causes of cardiopulmonary arrest (CPA).

According to the *Cardiomêtro* , an indicator of the number of deaths from CVD, created by the Brazilian Society of Cardiology, between 2004 and 2014, ischemic diseases constituted the group of cardiovascular causes with the highest prevalence of death events due to CVD.^4^The data in the literature regarding the incidence of CPA in Brazil are scarce, and the impact of this event on the mortality of individuals is observed.^5 - 7^

In this context, this study aims to describe the number of home deaths due to CPA notified by SAMU in the city of Belo Horizonte in 2020 and to compare the home deaths due to CRA in March 2020 in relation to March 2018 and March 2019.

## Methodology

This study is part of the SAMU notification service in Belo Horizonte, Minas Gerais, Brazil, and it refers to data collected in March 2018, March 2019, and March 2020. The notifications were selected based on the manual handling of files regarding the total attendance of the teams in the previously determined period. Exclusion criteria were not established for evaluating users/deaths. Thus, sampling was performed for convenience, covering all notifications registered with the service during the periods described.

This is a retrospective observational study carried out based on the analysis of primary data on mortality due to CPA in citizens assisted by SAMU in Belo Horizonte.

### Statistical Analysis

Age, occurrence characteristics (day of the month and time of day) and clinical characteristics (cause of CPA, medical follow-up, and associated comorbidities) were collected. The mortality rate was calculated according to the SAMU notification system.

The data were collected by the researchers of the service and subsequently submitted to descriptive analysis. The descriptive analysis of the variables was performed using the distribution of frequencies and absolute numbers of the categorical variables. The prevalence of outcomes and 95% confidence intervals were estimated for the population.

For data analysis, the public and free OpenEpi® statistical program was used. Categorical variables were analyzed using frequency distribution and compared using the chi-square test. The significance level was set at 95% (p < 0.05).

## Results

A total of 1,662 deaths were recorded by SAMU in the month of March in 2018 (n = 563), 2019 (n = 494), and 2020 (n = 605). During this period (March), SAMU reported 919 home deaths due to CPA in the years 2018, 2019, and 2020, distributed in 260 deaths in March 2018 (28.3%), 283 deaths in March 2019 (30.8%), and 376 deaths in March 2020 (40.9%). It was observed that the death rates for the total number of attendances at SAMU during these periods were 0.51, 0.57 and 0.62, respectively.

Figure 1 shows a 33% increase in cases of home deaths between March 2018 and March 2020. Table 1 compares the gross number of deaths by CPA and other causes, showing that 2020 had more notifications of home deaths due to CPA, with statistical difference in relation to 2018 and 2019.

**Figure 1:** – Prevalence of death outcome in patients assisted by the Mobile Emergency Care Service of Belo Horizonte, Minas Gerais, Brazil, March 2018-2020.

**Table 1:** – Comparison of home deaths due to cardiopulmonary arrest and other causes in March 2018 and 2019 compared to March 2020, notified by the Mobile Emergency Care Service of Belo Horizonte, Minas Gerais, Brazil

Deaths	Years		p value *	Years		p value *
	2018	2020		2019	2020	
CPA	260	376	<0.01	283	376	0.05
Other causes	303	229		211	229	

CPA: cardiopulmonary arrest; p value* - chi-square Test.

Table 2 describes social, clinical, and notification characteristics. Of the deaths reported in March 2020, 63.8% of the patients were aged 60 years or over. According to the notification time, 63.7% occurred during the daytime with 37.7% in the morning and 26.0% in the afternoon, with no difference between the proportions of the 31 consecutive days of that period ( Table 2 ).

**Table 2:** – Descriptive analysis of deaths by cardiopulmonary arrest in March 2020, notified by the Mobile Emergency Care Service of Belo Horizonte, Minas Gerais, Brazil

Variables	n	%
**Social characteristics**		
**Age (years)**		
≤ 19	10	2.70
20-59	126	33.50
≥ 60	240	63.80
**SAMU occurrence characteristics**		
**Attendance**		
First half of March	190	50.50
Second half of March	186	49.50
**SAMU occurrence time**		
6:00 am to 11:59 am	142	37.70
12:00 pm to 5:59 am	98	26.00
6:00 pm to 11:59 pm	89	23.60
12:00 am to 05:59 am	47	12.70
**Clinical characteristics**		
**Cause of CPA**		
Clinical	326	86.70
Trauma	50	13.30
**Medical follow-up**		
Yes	45	12.00
No	331	88.00

*CPA: cardiopulmonary arrest; SAMU: Mobile Emergency Care Service; am: ante meridiem; pm: post meridiem. Sample size = 376.*

Regarding clinical characteristics, it was observed that approximately 87.0% of patients with CPA had other clinical comorbidities and that the majority of the evaluated sample did not have any medical follow-up according to the data collected (88.7%) ( Table 2 ). However, many of the patients’ companions and family were unaware of the patients’ history.

Table 3 describes clinical comorbidities notified by the service, which represents 87.0% (n = 331) of the patients evaluated, according to the course of the disease observed. Among the chronic diseases observed, hypertension was associated with CPA in 22.87% of the reported cases; heart failure and diabetes mellitus were present in 13.03% and 11.0% of cases, respectively. Among other comorbidities, it is interesting to note that in 38.4% of the reported cases, although the family or friends reported the presence of associated comorbidity, they were unable to specify the patient’s associated comorbidities.

**Table 3:** – Comorbidities related to deaths by cardiopulmonary arrest in March 2020, notified by the Mobile Emergency Care Service of Belo Horizonte, Minas Gerais, Brazil

Clinical comorbidities	n	%
Not reported	145	38.56
Arterial hypertension	86	22.87
Heart failure	49	13.03
Diabetes mellitus	42	11.17
Cancer	40	10.64
Dementia	26	6.91
Respiratory infections	23	6.12
Stroke	19	5.05
Arrhythmia	12	3.19
Urinary tract infection	3	0.80

*Sample size = 376.*

## Discussion

As a main result of this study, a gradual numerical increase was observed in the rate of home deaths due to CPA for the total number of attendances by SAMU and a proportional increase of 33% of home deaths in March 2020, which is the month when the World Health Organization declared the COVID-19 pandemic.^8^

Since the confirmation of the first case in Brazil, on February 26, 2020, the press, as well as health authorities, in the absence of vaccines or antiviral medications, has warned of the need for social distance, the use of masks, hand washing, and reinforcement for care regarding respiratory etiquette. In the city of Belo Horizonte, the first notification of COVID-19 occurred on March 16, 2020; however, since the world declaration of the pandemic, the city government instituted social isolation early, preventing residents from non-essential contact.

Approximately 80% of COVID-19 cases are mild or oligosymptomatic.^9^A recent study shows that 20% required hospital care, with 5% to 15% of these being treated in intensive care units requiring ventilatory support. Mortality for these patients can reach up to 80%.^9^SARS-CoV-2 can be transmitted from person to person (contact with hands, cough, or saliva droplets) or through surfaces and objects contaminated by the virus.^10^On May 16, 2020, more than 4,605,673 people had been diagnosed with COVID-19, and there were 310,180 deaths worldwide. In Brazil, by May 16, 2020, 222,877 cases had been recorded, with 15,046 deaths.^11^Among the measures to prevent COVID-19 infection, individuals are recommended to clean their hands, surfaces, and objects with water and soap or hand sanitizer. Other indications include avoiding contact with eyes, nose, and mouth; permanent use of a facemask; maintaining healthy eating and sleep habits; and social isolation and distancing.^12^

The increase in the number of deaths, in the context of the pandemic, can aggravate users’ fear of leaving social isolation and seeking medical assistance and essential services. This could delay the demand for health services affecting the underlying disease. It is interesting to note that almost 89% of our sample did not have medical follow-up, and in a similar frequency (87%) the nature of the CPA was the clinical cause.

Gonzales-Olmo et al.,^13^in a recent study, with data from the population of Madrid, demonstrated high levels of individual self-perception of greater vulnerability in relation to COVID-19 infection when seeking dental care, which is considered an essential health service. Thus, the researchers observed that the sample of individuals over 60 years of age with systemic diseases avoided dental care most of the time.^13^

In accordance with our results, Holmes et al. (2020) indicated that there was a structural break in the time series of weekly admissions deferred annually according to emergency services in the United Kingdom between September 2019 and April 2020.^14^These researchers observed the period of time corresponding to September 2019 and April 2020.

Scales for assessing individuals’ fear have been developed and are in the validation phase (such as the “Fear of COVID-19 Scale”) in order to assess this emotion. New studies are expected to assess the context of the pandemic in this regard, monitoring fear at the expense of not seeking essential health services, improving quality of life, and delaying mortality due to causes that do not involve COVID-19.^15 - 17^

Julia et al.^18^report on the reorganization of the health service in France during the pandemic. Important investments have been made in terms of teleconsultations for the follow-up of patients with COVID-19, but the most vulnerable population with difficulties in accessing the internet and digital technologies or those with language barriers were left without adequate assistance. In addition, the available hospital beds have been drastically reduced, and primary health care professionals have thus had to deal with emergencies due to chronic diseases.^18^Home visits have been divided according to patients with and without COVID-19, leading to service overload. A flow for primary care has now been adopted for patients with chronic diseases, in order to avoid delays for outpatient control.^17^

In Belo Horizonte, there has been a reduction in demand from the population for care in the Basic Health Units and the Emergency Care Units. During the first four months of 2019, 1,478,905 people were attended; during the same period of 2020, this number was 1,215,543, which represents an 18% reduction.^19^Thus, it is important to monitor the management of chronic diseases and promote educational actions so that the population understands the importance of health care follow-up and the regular use of necessary medicines. Accordingly, the Municipal Health Department of Belo Horizonte has updated the flow of care in primary health care.^19 , 20^

Souza et al.^21^report on the primary health care provided by SUS in Brazil and on the investments that have been destined for acquisition of equipment and expansion of hospital beds for patients with COVID-19. They reinforce the need to strengthen primary care as an instrument to prevent collapse in the health system, avoiding deaths due to COVID-19 and chronic diseases.^21^It is necessary to reflect on how to find the balance so that health actions are not paralyzed during the pandemic.

The Municipal Health Department of Belo Horizonte has carried out initiatives such as training through web conferences, web classes, and discussion of clinical cases and technical notes for care and flow of patients with COVID-19, establishing partnerships for online care and assisting patients risk groups. At the same time, it has been taking measures to adapt emergency services and primary health care, seeking to avoid overloading services and promoting better care for patients with and without COVID-19.^20^

The increased numbers of home deaths have aroused the attention of health managers, and the idea is to reinforce the importance of outpatient control of chronic diseases and to clarify adopted safety measures to the population regarding clinical complications not related to COVID-19 during the pandemic.

It is a favorable moment to reinvent the relationship between users and primary health care professionals, seeking greater closeness to establish bonds, strengthen the importance of controlling chronic diseases and avoiding unnecessary deaths. It is also necessary to implement measures to improve death surveillance. Primary health care services should organize assistance and patient flows in order to ensure adequate care for patients with and without COVID-19.

As a study limitation, we point out the high percentage of individuals for whom it was not possible to collect information about the existence of comorbidities (38.5% of the sample) due to the lack of information from the interviewed relatives or friends, which may have underestimated the values presented.

## Conclusion

The results point to an increase in the number of home deaths due to CPA notified by SAMU in March 2020 in relation to March 2018 and March 2019 in Belo Horizonte, Minas Gerais. It is necessary to carry out new studies with a longitudinal design in order to monitor the increase in mortality of health system users and analyze its causal relationships in order to avoid deaths due to other diseases that do not involve COVID-19.
